# Electroconvulsive therapy electrode placement for bipolar state-related targeted engagement

**DOI:** 10.1186/s40345-019-0146-z

**Published:** 2019-05-04

**Authors:** Christopher C. Abbott, Jeremy Miller, Megan Lloyd, Mauricio Tohen

**Affiliations:** Department of Psychiatry & Behavioral Sciences, University of New Mexico Health Sciences Center, University of New Mexico School of Medicine, Albuquerque, NM 87131 USA

**Keywords:** Bipolar disorder, Electroconvulsive therapy, Electrode placement

## Abstract

**Background:**

Electroconvulsive therapy (ECT) is an effective treatment for all bipolar states. However, ECT remains underutilized, likely stemming from stigma and the risk of neurocognitive impairment. Neuroimaging research has identified state-specific areas of aberrant brain activity that may serve as targets for therapeutic brain stimulation. Electrode placement determines the geometry of the electric field and can be either non-focal (bitemporal) or more focal (right unilateral or bifrontal). Previous research has shown that electrode placement can impact clinical and cognitive outcomes independent of seizure activity. This review critically examines the evidence that focal (unilateral or bifrontal) electrode placements target specific aberrant circuitry in specific bipolar states to optimize clinical outcomes. We hypothesize that optimal target engagement for a bipolar state will be associated with equivalent efficacy relative to bitemporal non-focal stimulation with less neurocognitive impairment.

**Methods:**

We performed a literature search in the PubMed database. Inclusion criteria included prospective, longitudinal investigations during the ECT series with specific electrode placements within a bipolar state from 2000 to 2018.

**Results:**

We identified investigations that met our inclusion criteria with bipolar mania (n = 6), depression (n = 6), mixed (n = 3) and catatonia (n = 1) states. These studies included clinical outcomes and several included cognitive outcomes, which were discussed separately.

**Conclusions:**

While the heterogeneity of the studies makes comparisons difficult, important patterns included the reduced cognitive side effects, faster rate of response, and equivalent efficacy rates of the focal electrode placements (right unilateral and bifrontal) when compared to non-focal (bitemporal) placement. Further avenues for research include more robust cognitive assessments to separate procedure-related and state-related impairment. In addition, future studies could investigate novel electrode configurations with more specific target engagement for different bipolar states.

## Introduction

Treatment resistance to pharmacotherapy in bipolar disorder is unfortunately common across all phases of the illness (Thirthalli et al. 2012). Treatment resistance to pharmacotherapy is dependent on the phase of illness and has multiple definitions and thresholds. In the context of bipolar depression, treatment resistance is commonly defined as a failure of two antidepressant trials with concurrent treatment with a mood stabilizer (Gitlin 2006). Despite optimal treatment in the Systematic Treatment Enhancement Program for Bipolar Disorder (STEP-BD), 25% of the bipolar subjects failed to achieve symptom remission in 2 years of follow-up (Perlis et al. 2006). Treatment resistance and acuity are common indications for electroconvulsive therapy (ECT) in bipolar patients. ECT is effective in mania, mixed, and depressed states of bipolar disorder as well as maintenance phases of treatment (Medda et al. 2014). Despite the overwhelming effectiveness as a true mood stabilizer, patients and clinicians do not consider ECT earlier in the treatment algorithm because of the risk of neurocognitive impairment (Thirthalli et al. 2012; MacQueen et al. 2007; UK ECT Review Group 2003). The risk of ECT-mediated neurocognitive impairment is related to both patient (age, medical comorbidities, cognitive reserve) and treatment related factors (electrode placement, stimulation parameters, treatment number and frequency) (McClintock et al. 2014). The risk and benefit ratio may be optimized by individualizing ECT treatment to maximize mood stabilizing properties while minimizing neurocognitive impairment.

While the exact mechanism of action remains elusive, the therapeutic components of ECT include electric stimulation and generalized seizure activity. Electrode placement dictates the geometry of the electric field and, in conjunction with pulse amplitude, determines the percentage of activated brain volume (Peterchev et al. 2010). The anatomic location and amount of electric field strength may be related to clinical outcomes (Abbott et al. 2016). Bitemporal (BT) electrode placement is the oldest and most effective electrode placement for depressive episodes with respect to clinical efficacy and speed of response (Kellner et al. 2010). BT electrode placement is also the most non-focal method of stimulation and is associated with the greatest risk of neurocognitive impairment (Semkovska et al. 2011). Stimulation of the medial temporal lobes, independent of generalized seizure activity, is associated with increased risk of neurocognitive impairment and prompted efforts to develop more focal methods of stimulation that spare the hippocampus (Nobler et al. 2001; Sackeim et al. 1996; Nahas et al. 2013). For example, bifrontal and right unilateral electrode placement have more targeted engagement to the frontal and non-dominant hemisphere, respectively, relative to the non-focal bitemporal electrode placements.

The more focal electrode placements have unique patterns of brain stimulation that may have differential effectiveness in specific bipolar states. The older lateralization hypothesis of mood control suggests that left hemisphere stimulation may be effective in treating mania (Sackeim et al. 1982). More recent neuroimaging investigations have not confirmed state-related differences in laterality but have identified several candidate circuits for targeted engagement within the corticolimbic model of bipolar disorder (Blumberg et al. 2003; Strakowski et al. 2011; Brooks and Vizueta 2014; Strakowski et al. 2016). Critically, the large-scale networks such as sensorimotor and default mode networks may have unique patterns for depressive and manic phases of bipolar disorder (Martino et al. 2016). Extending state-related neuroimaging biomarkers to different bipolar states suggests that matching the stimulation pattern of the more focal electrode placements (unilateral or bifrontal) with the bipolar state should improve clinical outcomes (match the effectiveness of bitemporal electrode placement with reduced cognitive impairment). Limited evidence suggests that bifrontal electrode placement and prefrontal targeted engagement may have improved efficacy for mania (Blumenfeld et al. 2003). In contrast, right unilateral electrode placement may be ineffective for mania (Small 1985; Small et al. 1985; Milstein et al. 1987) but effective for depressive states with medial temporal lobe engagement (Abbott et al. 2014a). Thus, state-related electrode placements for optimal targeted engagement has the potential to optimize the risk/benefit ratio for ECT in bipolar disorder. However, neuroimaging has yet to confirm targeted engagement with specific electrode placements and bipolar states despite ample evidence with unipolar depression subjects (and combined unipolar and bipolar subjects) (Abbott et al. 2014b). Furthermore, the relative ineffectiveness of right unilateral electrode placement (and medial temporal lobe engagement) for mania is controversial and is based on older studies that did not utilize supra-threshold charge required for improved efficacy with unilateral electrode placements.

### Objectives of this review

Efforts to develop more focal stimulation are contingent on identification of state-related anatomic regions for target engagement. Here, we explore the existing evidence of differences in clinical outcomes among three different electrode placements (bitemporal, right unilateral, or bifrontal) for different bipolar states (mania, depressed, mixed, or catatonic episodes). We focused on prospective investigations and highlight the few studies that compared different electrode placements within a specific bipolar state. We hypothesized that the more focal electrode placement (e.g. bifrontal, right unilateral) would target the state-specific neuroanatomic structures implicated in the pathology of a bipolar state (prefrontal cortices for mania, medial temporal lobes for depression), thus maintaining efficacy of bitemporal electrode placement while decreasing the risk of neurocognitive impairment.

## Methods

We conducted a PubMed search with the following MeSH terms: “Bipolar Disorder/therapy”[MAJR]) AND “Electroconvulsive Therapy/methods”[MAJR] between January 1, 2000 and December 31, 2018. We selected this date range to acknowledge the development of clinically effective right unilateral supra-threshold (6x’s seizure threshold treatments) (Sackeim et al. 2000). Unilateral ECT investigations prior to this date used sub-optimal dosing (seizure threshold or low dose) with right unilateral ECT. Inclusion criterion included prospective investigations that included information on bipolar state (manic, mixed, depressed, subsyndromal, and catatonic) and electrode placement (bitemporal, right unilateral and bifrontal). Exclusion criteria included the following: (1) investigations that did not separate bipolar and unipolar depression subjects, (2) investigations focused exclusively on the maintenance phase of treatment, (3) investigations that did not include a final assessment at the end of the ECT series, and (4) review articles, case reports or case series. The exclusion of maintenance ECT from this search strategy was based on the role of state-specific targeted engagement that may not be applicable to maintenance phases of treatment. *Identification:* Our initial PubMed search identified 106 investigations. *Screening*: We reviewed abstracts and titles to select 24 for detailed review. We excluded two ECT bipolar mania investigation: a retrospective chart review (Thirthalli et al. 2009) and a case series with no summary statistics (Anand 2016). A previous meta-analysis (Dierckx et al. 2012) concluded that ECT was equally effective in bipolar and unipolar depression; however, it did not include electrode placements (Dierckx et al. 2012). We excluded one investigation that did not include information on electrode placement in bipolar and unipolar depression (Prudic et al. 2004). Two investigations were excluded as retrospective chart reviews (Grunhaus et al. 2002; Hallam et al. 2009). Three investigations did not separate bipolar and unipolar depressed subjects (Heikman et al. 2002; Bailine et al. 2000; Ranjkesh et al. 2005). *Eligibility and Identification:* We finalized our review with the selection of 14 ECT bipolar investigations for qualitative synthesis [one investigation reported clinical and cognitive results separately (Kessler et al. 2014; Schoeyen et al. 2015) and another large investigation reported results from all bipolar states (Perugi et al. 2017)]. We organized the results of our review based on bipolar state and highlight the investigations that directly compared electrode placements.

## Results: ECT electrode placement and bipolar phase

**Fig. 1:**
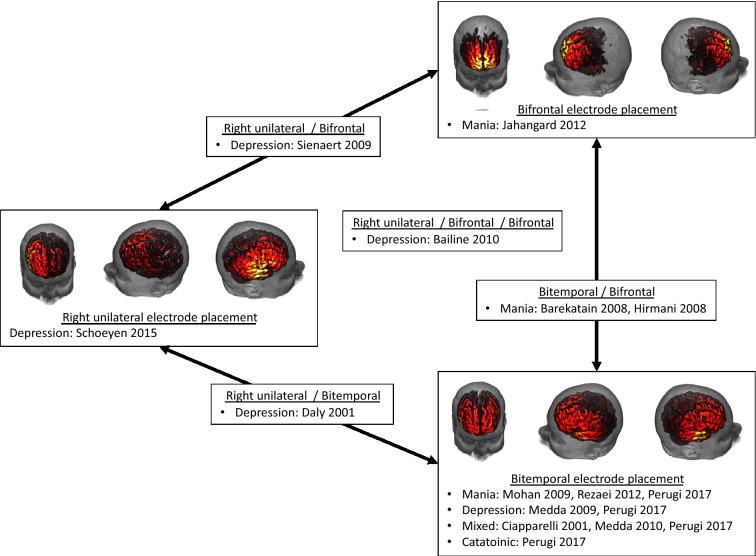
Electric field modeling for bitemporal, right unilateral and bifrontal electrode placements with the associated studies included in this review. The electric field is modeled for 800 milliamperes with a threshold of 0.35 V/cm (threshold for neuronal firing)

We have summarized the included investigations and representative electric fields for bitemporal, bifrontal and right unilateral electrode placements (single subject, 800 milliampere pulse amplitude) in Fig. 1.We have also summarized our findings in Tables 1, 2, 3 and 4.

**Table 1:** Electrode placement and bipolar mania

	Electrode placement, study design	N (age ± SD)	Stimulation parameters	Clinical assessment	Clinical outcome
Electrode placement comparison					
Barekatain et al. (2008)	BT, seizure threshold	14 (27.4 ± 9)	1 ms PW, 900 mA, thrice weekly	Young Mania Rating Scale, response criteria > 50% improvement	No significant difference between groups; all completers met response criteria
	BF, 1.5 times seizure threshold	14 (23.7 ± 4)			
Hiremani et al. (2008)	BT, 1.5 times seizure threshold	19 (28.7 ± 9)	1.5 × ST, 1.5 ms PW, 800 mA, thrice weekly	Young Mania Rating Scale	No significant group differences (88% response for BF, 72% for BT) but BF had a faster response evident by the third ECT treatment
	BF, 1.5 times seizure threshold	17 (25.8 ± 9)			
No electrode placement comparison					
Mohan et al. (2009)	BT, seizure threshold	26 (28.1 ± 7)	1.5 ms pulse width, 800 mA current amplitude, twice weekly	Young Mania Rating Scale, remission < 10	23/26 (88.5%) remitted
	BT, 2.5 times seizure threshold	24 (25.7 ± 7)			21/24 (87.5%) remitted
Jahangard et al. (2012)	BF, 1.5 times seizure threshold with sodium valproate	21 (32.8 ± 10)	Parameters not documented, thrice weekly	Young Mania Rating Scale, Primary assessment completed after 6th ECT treatment	Pre-ECT YMRS = 33 ± 10 to 6th-ECT YMRS = 19 ± 7
	BF, 1.5 times seizure threshold without sodium valproate	21 (31.43 ± 9)			Pre-ECT YMRS = 33 ± 5 to 6th-ECT YMRS = 24 ± 6 (no group differences)
Rezaei et al. (2012)	BT, pre-medicated with remifentanil	15 BT (30.8 ± 9)	1 ms pulse width, other parameters not documented	Young Mania Rating Scale	Pre-ECT YMRS = 29 ± 8 to end-ECT YMRS = 10 ± 9
	BT, pre-medicated with saline	14 BT (29.2 ± 9)			Pre-ECT YMRS = 28 ± 6 to End-ECT YMRS = 13.3 ± 8 (no group differences)
Perugi et al. (2017)	BT, age-based algorithm, community setting	8 BT (41.0 ± 11)	1 ms, pulse width 1.5–4 ms, 800 mA, twice weekly	Clinical Global Impression Improvement Scale, responder ≤ 2	75% response rate

**Table 2:** Electrode placement and bipolar depression

	Electrode placement, study design	N (age ± SD)	Stimulation parameters	Clinical assessment	Clinical outcome
Electrode placement comparison					
Daly et al. (2001) (studies 2 and 3 with supra-threshold stimulations for RUL and BT)	RUL, six times threshold, bipolar and unipolar depression	14 unipolar depression (overall age: 59 ± 15), 6 bipolar depression (overall age: 56 ± 16)	1.5 ms pulse width, 800 mA pulse amplitude, thrice weekly	Hamilton Depression Rating Scale, initial response rate 60% reduction and maximal score of 16	Initial response rate: 71% for unipolar depression (9 ± 1 treatments), 100% for bipolar depression (7 ± 2 treatments)
	BT, 1.5 times seizure threshold, bipolar and unipolar depression	33 unipolar depression, 14 bipolar depression			Initial response rate: 73% for unipolar depression (9 ± 2 treatments), 86% for bipolar depression (8 ± 2 treatments)
Sienaert et al. (2009)	RUL, 1.5 times seizure threshold	51 unipolar depression (55 ± 12), 13 bipolar depression (55 ± 13) randomized to either RUL or BF	0.3 ms pulse width, 800 mA pulse amplitude, twice weekly	Hamilton Depression Rating Scale, moderate remission criteria < 10	Response and remission rates did not differ by diagnosis (unipolar or bipolar diagnosis) or electrode placement; bipolar patients had a faster rate of response, which was independent of electrode placement
	BF, six times seizure threshold				
Bailine et al. (2010)	RUL, six times threshold; BF, 1.5 times seizure threshold, and BT, 1.5 times seizure threshold	170 unipolar depression (55 ± 16) and 50 bipolar depression (49 ± 13)	900 mA, thrice weekly	Hamilton Depression Rating Scale, moderate remission criteria < 10	EP and diagnosis were not associated with study outcomes (continuous measure with HDRS or remission criteria)
No electrode placement comparison					
Medda et al. (2009)	BT, unipolar depression	17 (54 ± 17)	Age-based settings, 800 mA, pulse width not documented, twice weekly	Hamilton Depression Rating Scale, remission < 8	12/17 (71%) Remission
	BT, bipolar I mre depressed	46 (51 ± 12)			16/46 (35%) Remission (lower remission rates relative to unipolar depression)
	BT, bipolar II mre depressed	67 (53 ± 14)			29/67 (43%) Remission (lower remission rates relative to unipolar depression)
Schoeyen et al. (2015) (clinical results)	RUL ECT, bipolar I and II	38 (48 ± 10)	Age-based settings, both Thymatron and Mecta ECT devices, 0.5 ms pulse width, thrice weekly	Montgomery-Asberg Depression Rating Scale, remission < 12	Mean MADRS score 15 ± 7 (improved relative to pharmacotherapy), response rate 74% (improved relative to pharmacotherapy), and remission rate 35% (no difference relative to pharmacotherapy)
	Pharmacotherapy, bipolar I and II	35 (48 ± 13)	Algorithm-based pharmacological treatment		Mean MADRS score 20 ± 10, response rate 35%, remission rate 30%
Perugi et al. (2017)	BT, age-based algorithm	295 (49.8 ± 13)	1 ms, pulse width 1.5–4 ms, 800 mA, twice weekly	Clinical Global Impression Improvement Scale, responder ≤ 2	68.1% responders

**Table 3:** Electrode placement and bipolar mixed and bipolar catatonia

	Electrode placement, study design	N (age ± SD)	Stimulation parameters	Clinical assessment	Clinical outcome
Mixed episodes					
Ciapparelli et al. (2001)	BT, bipolar mixed	41 (38 ± 12)	1 to 2 ms pulse width, 550 to 800 mA current amplitude, twice weekly	Montgomery-Asberg Depression Rating Scale (response defined as > 50% reduction), Brief Psychiatric Rating Scale, and Clinical Global Impression (response defined as ≤ ”mildly ill”)	CGI response criteria = 56%, MADRS response criteria = 78%, higher response rates for mixed episode subjects
	BT, bipolar depressed	23 (41 ± 14)			CGI response criteria = 26%, MADRS response criteria = 52%
Medda et al. (2010)	BT, bipolar mixed	50 (39 ± 13)	Age-based formula, 1.0 ms pulse width, 800 mA current amplitude, twice weekly	Hamilton Depression Rating Scale (response defined as > 50% reduction, remission ≤ 8), Brief Psychiatric Rating Scale, Young Mania Rating Scale, and Clinical Global Improvement Scale (response defined as ≤ ”much improved”, remission rate as ≤ ”very much improved”)	CGI response criteria = 76%, similar response and remission rates among both groups irrespective of rating scale
	BT, bipolar depressed	46 (51 ± 12)			CGI response criteria = 67%
Perugi et al. (2017)	BT, age-based algorithm	197 (44 ± 13)	1 ms, pulse width 1.5–4 ms, 800 mA, twice weekly	Clinical Global Impression Improvement Scale, responder ≤ 2	72.9% responders
Catatonic episodes					
Perugi et al. (2017)	BT, age-based algorithm	26 (49.50 ± 13)	1 ms, pulse width 1.5–4 ms, 800 mA, twice weekly	Clinical Global Impression Improvement Scale, responder ≤ 2	80.8% responders

**Table 4:** Electrode placement and cognition

	Electrode placement	Cognitive assessment	Cognitive outcome
Mania			
Mohan et al. (2009)	BT	Mini Mental State Exam, Wechsler Memory Scale, Scale for autobiographical memory	MMSE and WMS scores declined in both groups, but no group differences between seizure threshold and 2.5 times seizure threshold
Rezaei et al. (2012)	BT	Mini Mental State Exam, Reorientation time	MMSE scores declined in both groups (± remifentanil), but no group differences
Barekatain et al. (2008)	BT and BF	Mini Mental State Exam	BF group had higher MMSE scores after the 6th and final ECT treatment
Hiremani et al. (2008)	BT and BF	Trail Making Test, Verbal Fluency Test (Category), Paired Associate Learning Test, and Complex Figure Test (completed after fifth ECT treatment)	No group differences
Depression			
Kessler et al. (2014)	RUL (and pharmacotherapy)	MATRICS Consensus Cognitive Battery, Autobiographical Memory Interview-Short Form	Both groups demonstrated improvement in every domain of MCCB with no group by time interaction; AMI-SF had a group by time interaction indication reduced autobiographical memory consistency in the ECT arm

### Bipolar Mania

#### Electrode placement comparisons

Two investigations compared bitemporal with bifrontal electrode placement (Hiremani et al. 2008; Barekatain et al. 2008). Although both investigations included small samples (< 20), the results demonstrated similar effectiveness with both bifrontal and bitemporal electrode placements. Barekatain et al. (2008) compared bifrontal at 1.5 times seizure threshold and bitemporal at seizure threshold. Post-ECT YMRS scores were equivalent for both arms, but attrition was notable in this small study with only 8 of 14 (57%) subjects in the bitemporal arm and 10 of 14 subjects (71%) in the bifrontal arm completing the study. Hiremani et al. (2008) compared bifrontal at 1.5 times seizure threshold and bitemporal at 1.5 times seizure threshold. BF had a faster rate of response evident by the third ECT treatment, but the overall response rates were equivalent (88% response for bifrontal, 72% for bitemporal).

#### No electrode placement comparisons

Four investigations used one electrode placement (either bitemporal or bifrontal) and focused on stimulus intensities, concurrent psychotropic medications, and anesthetic augmentation strategies. One investigation used bitemporal electrode placement and compared two different stimulation intensities in subjects with bipolar mania with brief pulse ECT (1.5 ms) (Mohan et al. 2009). This investigation had the largest sample size for ECT-mania with 26 subjects in the “seizure threshold” and 24 subjects in the “2.5 times seizure threshold” groups. The primary outcome was the Young Mania Rating Scale (YMRS). The results demonstrated very high and equivalent remission rates (88%) in each arm. The second investigation to use bitemporal electrode placement compared two groups randomized to remifentanil or normal saline to improve clinical outcomes (Rezaei et al. 2012). Overall response rates were not recorded, but both arms demonstrated an equivalent reduction in YMRS. The third bitemporal ECT investigation assessed response rates from a community sample and demonstrated a 75% response rate (Perugi et al. 2017). The only study to focus exclusively on bifrontal electrode placement in mania compared two groups randomized to continue or discontinue sodium valproate (Jahangard et al. 2012). The two groups had an equivalent reduction in YMRS at the “primary” mid-series assessment (after the sixth ECT treatment) but notable attrition (69%) by the end of the ECT series.

#### Summary

We are unable to make definitive comparisons between the investigations focused on a single electrode placement due to attrition and inconsistent reporting of clinical ratings. However, both bifrontal and bitemporal electrode placements appear to be effective for mania independent of alternative study aims (comparison of stimulation intensities or pharmacological augmentation strategies). Notably, bifrontal may be associated with a faster rate of response relative to bitemporal electrode placement, but larger studies are needed to prove this relationship.

### Bipolar Depression

#### Electrode placement comparisons

Three investigations compared differences in clinical outcomes among bipolar patients with different electrode placements (Daly et al. 2001; Sienaert et al. 2009; Bailine et al. 2010). Daly et al. (2001) focused on the dose-finding studies with right unilateral and bitemporal electrode placements with different stimulation intensities (right unilateral from seizure threshold to six times threshold, bitemporal from seizure threshold to 2.5 times threshold). For purposes of this review, we focused on the results for the supra-threshold stimulations: right unilateral at six times threshold and bitemporal at 2.5 times threshold. The sub-sample in this analysis was very small (n = 6 for right unilateral, n = 14 for bitemporal). The right unilateral supra-threshold treatments included a very small sample (n = 6) but 100% response and a faster rate of response for the bipolar depressed (7 ± 2 treatments) relative to the unipolar depressed subjects (9 ± 2). The bitemporal supra-threshold treatments also had a robust response (86%). Sienaert et al. (2009) assessed right unilateral and bifrontal electrode placement in a sample of unipolar and bipolar patients. Results did not reveal any diagnostic or electrode placement differences, but bipolar subjects had a faster rate of response. Bailine et al. (2010) assessed the efficacy of right unilateral, bifrontal, and bitemporal electrode placements in a large sample of unipolar (n = 170) and bipolar subjects (n = 50). Electrode placement and diagnosis (bipolar or unipolar depression) were not significant. Results from each electrode placement were not reported separately.

#### No electrode placement comparisons

Three investigations used one electrode placement (bitemporal or right unilateral) to establish outcomes in a large community sample (Perugi et al. 2017), compare differential outcomes across diagnostic categories (Medda et al. 2009), or differences in clinical outcomes relative to pharmacotherapy (Schoeyen et al. 2015). Medda et al. (2009) used bitemporal electrode placement to compare clinical outcomes across three different diagnostic categories: unipolar depression, bipolar I, and bipolar II subjects. The unipolar depressed group was the smallest group but had better rates of remission (12 subjects went to remission out of 17, 71% remission rate) relative to bipolar I (16/46, 35%) and bipolar II (29/67, 43%). Schoeyen et al. (2015) used a randomized controlled trial comparing right unilateral electrode placement (age-based formula) relative to pharmacotherapy for bipolar depression. The right unilateral arm had more clinical improvement and higher response rates relative to the pharmacotherapy arm, but the remission rates (35% in the ECT arm) were similar. Perugi et al. (2017) conducted the largest study to date on bipolar and ECT (total n = 522, bipolar depression n = 295). The study used bitemporal electrode placement and found a high response rates in bipolar depression (68%, response defined as Clinical Global Impression Improvement Subscale (CGI) < 2 or “much improved”).

#### Summary

Perugi et al. (2017) established the rate of response with bipolar depression and bitemporal electrode placement at 68% with a large sample of ~ 300 subjects. The different outcome measures associated with each study (CGI in the Perugi investigation compared to traditional depression ratings with rigorous remission criteria) complicates study and electrode placement comparisons. The low remission rates in the Schoeyen investigation prompted debate regarding right unilateral electrode placement in ECT, but the remission rates are comparable to an earlier bitemporal investigation (Schoeyen et al. 2015; Medda et al. 2009; Kellner and Fink 2015; Kotzalidis et al. 2015). The investigations comparing different types of electrode placements (right unilateral, bifrontal and bitemporal) included both unipolar and bipolar subjects that may have been underpowered to detect differences in response or remission rates within the bipolar group (Daly et al. 2001; Sienaert et al. 2009; Bailine et al. 2010).

### Bipolar Mixed and Catatonia

We identified three investigations focused on ECT and bipolar mixed episodes (Perugi et al. 2017; Ciapparelli et al. 2001; Medda et al. 2010). We also identified one investigation that assessed ECT response with bipolar catatonia (Perugi et al. 2017). All investigations (for both bipolar mixed and catatonia) used bitemporal electrode placement and had similar efficacy irrespective of outcome measure. The initial efficacy favoring bipolar mixed relative to bipolar depression (Ciapparelli et al. 2001) was not replicated in a later study, which demonstrated similar, robust response rates for both depressed and mixed episodes (Medda et al. 2010). The bitemporal investigation with catatonia verified the expected high rate of response with this indication (Perugi et al. 2017).

### Bipolar state, Electrode Placement and Cogntion

#### Mania

Two investigations assessed ECT-mediated neurocognitive impairment with bitemporal electrode placement in subjects with mania (Mohan et al. 2009; Rezaei et al. 2012). Both investigations demonstrated decline in cognitive performance with the Mini Mental State Exam (Mohan et al. 2009; Rezaei et al. 2012) and the Weschler Memory Scale (Rezaei et al. 2012). Two investigations measured longitudinal cognitive performance in subjects receiving bifrontal and bitemporal electrode placements (Hiremani et al. 2008; Barekatain et al. 2008). Hiremani et al. (2008) included a thorough cognitive assessment but did not find group differences with bifrontal or bitemporal electrode placements. In contrast, Barekatain et al. (2008) found group differences after the sixth ECT treatment with improved cognitive scores in the bifrontal arm.

#### Depression

Only one investigation assessed longitudinal cognitive performance during ECT with bipolar depression. Kessler et al. (2014) assessed cognitive performance with the MATRICS Consensus Cognitive Battery, and Autobiographical Memory Interview-Short Form in the right unilateral ECT and pharmacotherapy arms. Both groups demonstrated improvement in every domain of the MATRICS Consensus Cognitive Battery. However, the right unilateral ECT arm had reduced consistency with the Autobiographical Memory Interview-Short Form.

#### Summary

Although the evidence is sparse, the more focal ECT electrode placements may be associated with less ECT mediated neurocognitive impairment irrespective of bipolar state. The conflicting results in the two bifrontal/bitemporal bipolar mania investigations may be attributed to the different assessment time points. The earlier cognitive assessment (after the fifth ECT treatment) had no differences between bitemporal and bifrontal electrode placements (Hiremani et al. 2008), but traditional end of ECT assessment resulted in group differences despite the use of a less sensitive cognitive measure (Barekatain et al. 2008). The right unilateral electrode placement in bipolar depression was associated with cognitive improvement on most measures and mirrored the cognitive results of pharmacotherapy arm (Kessler et al. 2014). The diminished consistency of the Autobiographical Memory Interview-Short Form must be interpreted in the context of the debatable psychometric properties of this test (Semkovska and McLoughlin 2013, 2014).

## Discussion

This review focused on the relationship of electrode placement on clinical outcomes in bipolar states. Bitemporal electrode placement is the most non-focal electrode placement and has higher efficacy rates relative to more focal bifrontal and right unilateral electrode placements. An adequate ECT trial should include bitemporal electrode placement in the context of non-response with focal electrode placements (Kellner and Fink 2015). Bitemporal electrode placement should also be considered the first-line electrode placement when acuity warrants rapid response (e.g., bipolar with catatonia) (Kellner et al. 2010; Perugi et al. 2017). However, the high efficacy rates seen in bitemporal electrode placement comes at a cost of increased risk of ECT-mediated neurocognitive impairment (Semkovska et al. 2011). We hypothesized that efficacy can be maintained, and cognitive risk reduced when more focal electrode placements are matched with state-related targeted engagement. The role of targeted engagement may result in different electrode placements for different bipolar states (e.g., bifrontal for mania, unilateral for depression). The available data is insufficient to support definitive conclusions, but several patterns emerged when reviewing the data. First, focal and non-focal electrode placements are effective for all bipolar states. Second, bifrontal electrode placement appears to have equivalent efficacy relative to bitemporal electrode placement for mania and is associated with less cognitive risk (Hiremani et al. 2008; Barekatain et al. 2008). Third, the more focal electrode placements reduce the cognitive risk across different states of bipolar disorder (Kessler et al. 2014; Barekatain et al. 2008).

Electrode placement is the most important stimulation variable development with respect to geometry of the electric field and electric field current path (Peterchev et al. 2010). To date, the focus on electrode placement development has been reduction of cognitive impairment as opposed to targeting specific state-related neuroanatomic targets (d’Elia 1970; Abrams and Taylor 1973). Novel electrode placements and stimulation methods were not included in this review but represent the next developmental step in improving the risk and benefit ratio for convulsive therapies. Focal Electrically Administered Seizure (FEAST) was recently implemented with a novel electrode placement consisting of a small anode over the right eyebrow and large cathode over the right motor cortex (Nahas et al. 2013; Chahine et al. 2014; Spellman et al. 2009). The intention was to concentrate the current flow to the right subcallosal cingulate and frontal poles and avoid the medial temporal lobes. Magnetic seizure therapy is another form of brain stimulation that uses magnetic pulses to induce seizure activity (Lisanby et al. 2001a, b, 2003). The magnetic stimulation reduces the anatomic variability relevant to electric fields and creates a focal but very superficial stimulation (Lisanby et al. 2003). The cognitive profile of magnetic seizure therapy appears promising, but the clinical efficacy of FEAST and magnetic seizure therapy has yet to be established in bipolar disorder (Cretaz et al. 2015). Other efforts to improve the focality of stimulated brain volume include low amplitude seizure therapy (Radman and Lisanby 2017; Youssef and Sidhom 2017), which has yet to be thoroughly investigated with bipolar disorder. Computer modeling of the ECT induced electric field has promised even more focal methods of stimulation with reduced electrode placement size and diminished distance between stimulation electrodes (Deng et al. 2013).

As the ability to increase the focality with magnetic or electric stimulation improves, the importance of selecting the optimal target region for a diagnosis, state, phenotype or individual becomes increasingly more important. While bitemporal electrode placement may maintain high efficacy rates across different diagnoses (major depressive disorder, bipolar disorder, schizophrenia) with greater risk of cognitive impairment, more focal stimulations may miss the optimal target region for a bipolar state and compromise the efficacy of the procedure (Cretaz et al. 2015). However, the premise of this review is that the efficacy of focal stimulations may be maintained if focused on the optimal anatomic target. This framework is illustrated in a recent investigation with transcranial magnetic stimulation. Sophisticated data-driven analysis methods combined neuroimaging with depression ratings to identify distinct depression “biotypes”. These biotypes successfully differentiated response to left dorsolateral or dorsomedial prefrontal cortical transcranial magnetic stimulation (Drysdale et al. 2017). Similar methods could be applied to a large sample of bipolar patients to identify phenotypes and areas of targeted engagement that may represent a more granular classification pattern than manic, depressed or mixed states.

The included investigations had several limitations that limit definitive conclusions from this review. First, the variability of clinical outcomes precluded formal statistical analysis. Second, the included studies were heterogeneous and included alternative outcomes not emphasized in this review (e.g., pharmacotherapy augmentation of ECT). Third, ECT parameters such as pulse width can impact both the efficacy as well as the cognitive risk of ECT but were not included in this review (Semkovska et al. 2011). Fourth, many of the included investigations relied on basic and insensitive cognitive screening measures to capture longitudinal changes in cognitive performance. Only a minority of the included studies included a thorough neuropsychological assessment (Kessler et al. 2014; Hiremani et al. 2008). Finally, seizure metrics (morphology, duration, post-ictal suppression) were not included but are related to the mood stabilizing properties of convulsive therapies.

## Conclusions

Recent prospective ECT investigations with different bipolar states (depressed, manic, mixed) have assessed clinical outcomes with different electrode placements. While not definitive, the more focal electrode placements do have the potential to have equivalent efficacy, expedite the rate of response, and reduce cognitive risk. These focal electrode placements may be specific for bipolar states (e.g., bifrontal for mania). However, serious gaps in the literature preclude formal guidelines as supra-threshold right (and left) unilateral electrode placements have not been adequately assessed in bipolar mania and remain controversial in the context of optimizing ECT outcomes for bipolar disorder. If prefrontal networks are specific to bipolar mania, then bifrontal electrode placement should prove to be more efficacious than the supra-threshold unilateral treatments, but this investigation has yet to be completed. In addition, optimal targeted engagement may be more granular than the bipolar state (manic, depressed, or mixed). For example, melancholic or atypical depressive episodes have unique phenomenology and may have different patterns of aberrant circuitry that ultimately require different patterns of stimulation similar to the aforementioned “biotype” research with transcranial magnetic stimulation. Future directions include the development of more focal brain stimulation that will challenge neuroimaging research to optimally define the phenotypically-defined region for targeted engagement. Furthermore, research with more thorough cognitive assessments to disentangle state-related cognitive impairment from procedure-related cognitive impairment is needed. Accurate, focal targeted engagement of the correct anatomic circuit for a given bipolar state will optimize clinical outcomes for bipolar disorder.
